# Comparative analysis of smart and traditional braces in orthodontic treatment efficiency

**DOI:** 10.6026/973206300213603

**Published:** 2025-10-31

**Authors:** Hiba Ikhlas Zain, Asifa C.K, Vanita Mansukhani, Sachin Katole, Kumari Pushpa, Mandar Pathak

**Affiliations:** 1 Private Practice, General Dentist, Moses Dental Clinic, Guruvayur, Thrissur, Kerala, India; 2 Department of Orthodontics and Dento Facial Orthopedics, Educare institute of Dental Sciences, Malappuram, Kerala, India; 3 Department of Conservative Dentistry and Endodontics, Sree Balaji Dental College, Velachery Rd, Narayanapuram, Pallikaranai, Chennai, Tamil Nadu 600100, India; 4 Department of Orthodontics, Saraswati Dhanwantari Dental College and Hospital, Parbhani, Parawa, Maharashtra, India; 5 Department of Orthodontics, Dr. B R Ambedkar College of Dental Sciences and Hospital, Patna, Bihar, India; 6 Department of Orthodontics, SDDC Parbhani, Parawa, Maharashtra, India

**Keywords:** Smart braces, traditional braces, orthodontic efficiency, treatment duration, patient satisfaction

## Abstract

Smart braces are a new orthodontic appliance using advanced materials and sensors for improved tooth movement. This study compared their efficiency, duration and patient satisfaction to traditional braces in a randomized hospital-based cohort of 120 patients. Data showed significantly shorter treatment times and fewer adjustments with smart braces. Patients also reported higher comfort and satisfaction levels. Thus, we show smart braces offer a more efficient and patient-friendly alternative to conventional orthodontic treatment.

## Background:

Orthodontic treatment is fundamentally based on correcting malocclusions by applying controlled mechanical forces and modifying the position of teeth within their alveolar bone [1]. Conventional braces (16-gauge stainless steel brackets and wires) have been the de facto gold standard of orthodontic treatment for many years [2]. While orthodontists have historically had success with conventional braces, the manual adjustments of the brackets and archwires often resulted in longer overall treatment times and an increase in chairside visits [3]. Recent advances in biomedical engineering have led to the development of "smart braces" that utilize shape-memory alloys, integrated sensors and digital tracking devices, to help with treatment. Smart braces can be designed to apply optimized forces and adapt in response to sensor feedback allowing the clinician to customize a treatment protocol based on the patient's unique needs [4, 5]. Smart braces also using biofeedback mechanisms and microcontrollers to view tooth movement in real-time, which may help mitigate overcorrection or the application of inefficient force. Incorporating these technologies is intended to reduce chair time, reduce human error, improve the precision and efficacy of tooth movement and ultimately reduce treatment time [6]. Besides the functional benefits, smart braces are typically manufactured with a focused-on aesthetics and patient comfort. Because smart braces are less visible and don't require frequent adjusting, this may provide more incentives for patient compliance and patient satisfaction throughout the treatment period [7]. Although smart braces provide these innovations, specific clinical evidence for the efficacy of using smart braces compared to conventional stainless-steel brackets is lacking. Specifically, systematic studies reporting treatment efficiency, force application, patient outcome and overall treatment time remain limited [8]. Therefore, it is of interest to compare the clinical efficacy of smart braces and conventional stainless-steel brackets for orthodontic treatment speciality, especially in terms of treatment duration, efficiency of tooth movement and patient satisfaction.

## Materials and Methods:

This was a prospective, randomized controlled study conducted at the Department of Orthodontics, ABC Medical College, over 18 months. A total of 120 patients aged 14 to 30 years with Class I or II malocclusions were randomly assigned into two groups: smart braces (Group A, n=60) and traditional braces (Group B, n=60). All patients underwent pre-treatment evaluation including cephalometric analysis and oral hygiene assessment. Treatment plans were standardized across groups. Smart braces included shape-memory alloy archwires and integrated sensor modules connected to a mobile app monitored by the orthodontist. Traditional braces used standard metal brackets and nickel-titanium wires. Monthly follow-ups were conducted and data on treatment duration, alignment scores using little's Irregularity Index (LII), number of adjustment visits and patient satisfaction (via questionnaire) were collected. Data were analyzed using SPSS v26 with significance set at p<0.05.

## Results:

The mean age of participants across both groups was 21.4 ± 4.3 years. Group A (smart braces) demonstrated a significantly shorter mean treatment duration (9.2 ± 1.1 months) compared to Group B (traditional braces) at 12.8 ± 1.4 months (p < 0.001). Little's Irregularity Index (LII) improved more rapidly in Group A at interim assessments, reflecting more efficient alignment. Group A also required fewer adjustment visits, with an average of 5.2 ± 0.8 compared to 9.6 ± 1.1 in Group B. Patient-reported satisfaction was notably higher in Group A, with a mean score of 8.6/10 versus 6.9/10 for Group B. These findings highlight the advantages of smart braces in reducing treatment duration, minimizing follow-up visits and improving patient satisfaction. Table 1 (see PDF) demonstrated significantly shorter treatment duration with smart braces compared to traditional braces. Table 2 (see PDF) showed that smart braces required fewer adjustment visits. Table 3 (see PDF) indicated that alignment, as measured by LII, improved more rapidly with smart braces at 6 months. Table 4 (see PDF) confirmed higher patient satisfaction among those treated with smart braces. Collectively, these results suggest that smart braces are more efficient, require less maintenance and provide a superior patient experience compared to traditional braces.

## Discussion:

This study demonstrated a statistically significant advantage of smart braces over traditional braces in terms of treatment duration, number of adjustment visits and patient-reported satisfaction [9]. The use of shape-memory alloys and real-time monitoring likely facilitated more consistent force application and timely interventions, aligning with prior findings by Chen *et al.* who reported faster orthodontic correction using thermally activated wires [10]. Smart systems also minimized the reliance on frequent manual adjustments, which may contribute to reduced chair-time and improved clinic efficiency [11]. Patient satisfaction, a critical component of orthodontic success, was higher in the smart brace group, potentially due to enhanced comfort, fewer appointments and faster visible results [12]. As per Koaban *et al.* the amalgamation of smart technology into orthodontic bracket systems is an exciting development. These brackets are equipped with sensors that allow for real-time data collection of the forces generated and applied to the teeth, enabling more precise adjustments and predictable outcomes. As the sensors monitor the magnitude as well as the direction of the forces, they give orthodontists significant insights for optimizing the treatment and contribute to better compliance in younger patients [13]. However, the cost of smart braces remains a limiting factor and long-term studies are needed to assess retention outcomes and relapse rates [14]. Nonetheless, this study supports their efficacy in routine orthodontic practice [15].

## Conclusion:

Smart braces offer improved treatment efficiency and greater patient satisfaction compared to traditional braces. Their incorporation into routine orthodontic practice may enhance outcomes, but further longitudinal studies are warranted.
